# Supplementary material for: Genetic influences on motor learning and superperformance mutants revealed by random mutational survey of mouse locomotion
Source: bioRxiv. 2023 Jun 29:2023.06.28.546756. Preprint. [Version 1] doi: 10.1101/2023.06.28.546756 (PMC10327015; doi:10.1101/2023.06.28.546756)
Supplement: Supplement 4 [file NIHPP2023.06.28.546756v1-supplement-4.pdf]

bioRxiv preprint doi: https://doi.org/10.1101/2023.06.28.546756; this version posted June 30, 2023. The copyright holder for this preprint (which was not certified by peer review) is the author/funder, who has granted bioRxiv a license to display the preprint in perpetuity. It is made available under aCC-BY 4.0 International license.

Supplementary information

Genetic influences on motor learning and superperformance mutants revealed by random mutational survey of mouse locomotion

Vikram Jakkamsetti [1], Qian Ma [1], Gustavo Angulo [1], William Scudder [1], Bruce Beutler [2, 3, 4, #], Juan M. Pascual [1, 5, 6, 7, #, *]

1 Rare Brain Disorders Program, Department of Neurology; 2 Center for Genetics of Host Defense; 3 Department of Immunology; 4 Department of Internal Medicine; 5 Department of Physiology; 6 Department of Pediatrics; 7 Eugene McDermott Center for Human Growild typeh & Development / Center for Human Genetics; The University of Texas Southwestern Medical Center, Dallas, Texas, USA

bioRxiv preprint doi: https://doi.org/10.1101/2023.06.28.546756; this version posted June 30, 2023. The copyright holder for this preprint (which was not certified by peer review) is the author/funder, who has granted bioRxiv a license to display the preprint in perpetuity. It is made available under aCC-BY 4.0 International license.

Supplementary figure 1

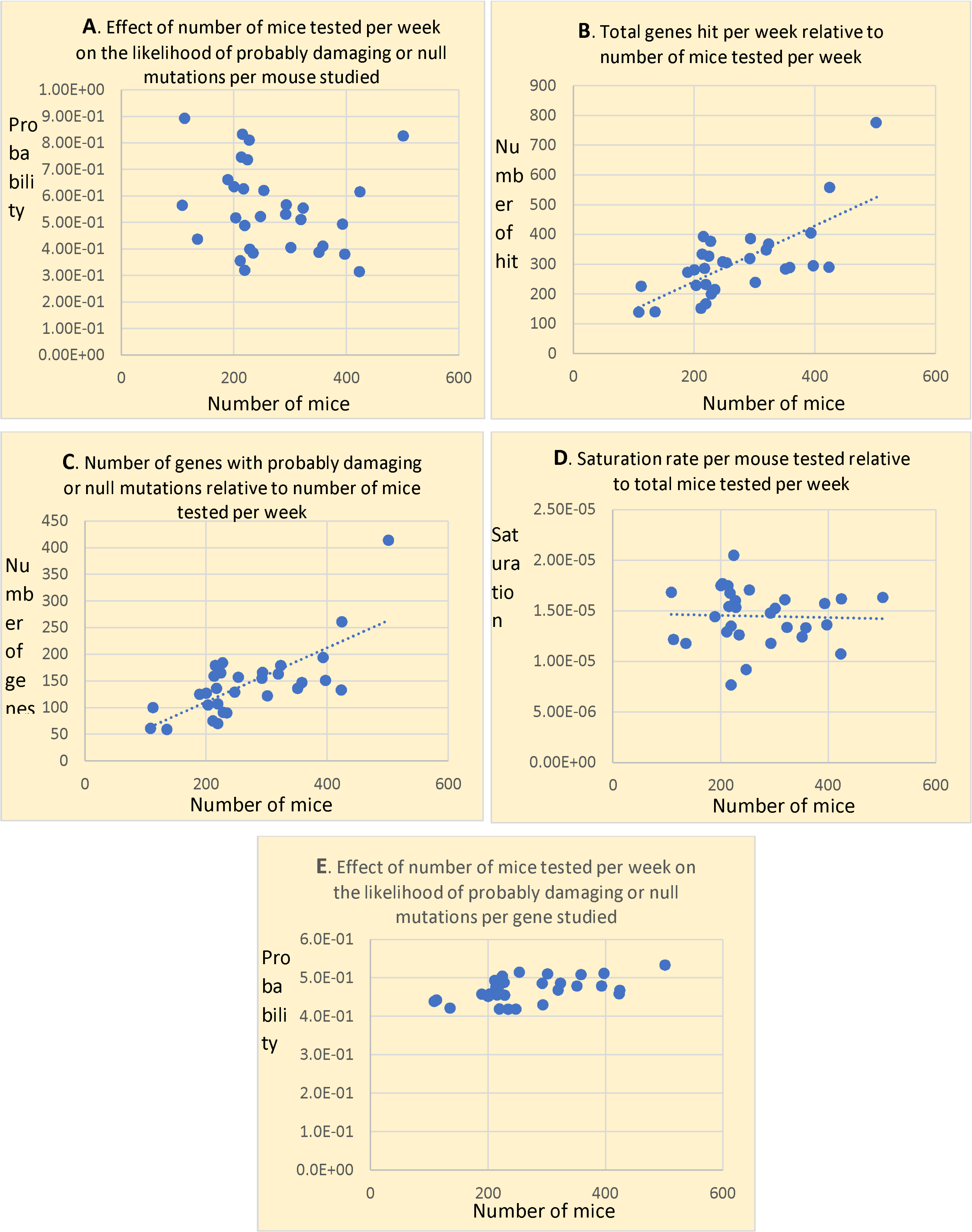

Efficiency of the motor screening. The genes affected include probably benign, possibly damaging, probably damaging and probably null mutations.

bioRxiv preprint doi: https://doi.org/10.1101/2023.06.28.546756; this version posted June 30, 2023. The copyright holder for this preprint (which was not certified by peer review) is the author/funder, who has granted bioRxiv a license to display the preprint in perpetuity. It is made available under aCC-BY 4.0 International license.

Supplementary figure 2

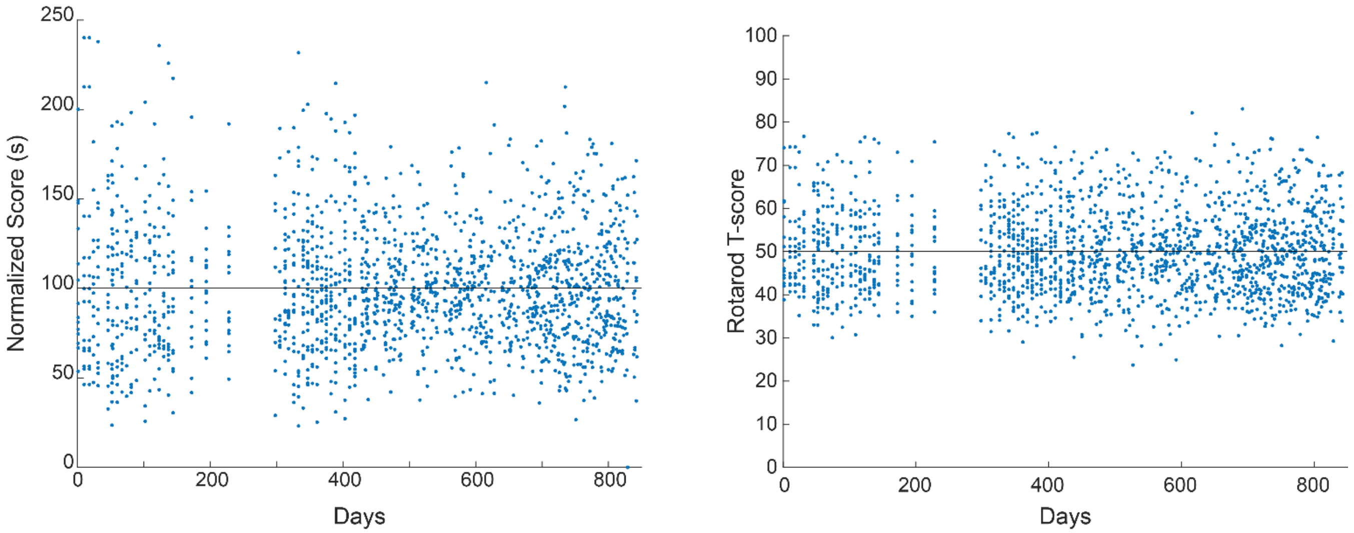

Normalized rotarod scores (left panel) and T-scores (right panel) of control wild type mice over time. Most rotarod T-scores lie within mean +/- two standard deviations (50 +/- 20) and almost all T-scores lie within 3 standard deviations of the mean (50 +/- 30).

bioRxiv preprint doi: https://doi.org/10.1101/2023.06.28.546756; this version posted June 30, 2023. The copyright holder for this preprint (which was not certified by peer review) is the author/funder, who has granted bioRxiv a license to display the preprint in perpetuity. It is made available under aCC-BY 4.0 International license.

Supplementary figure 3

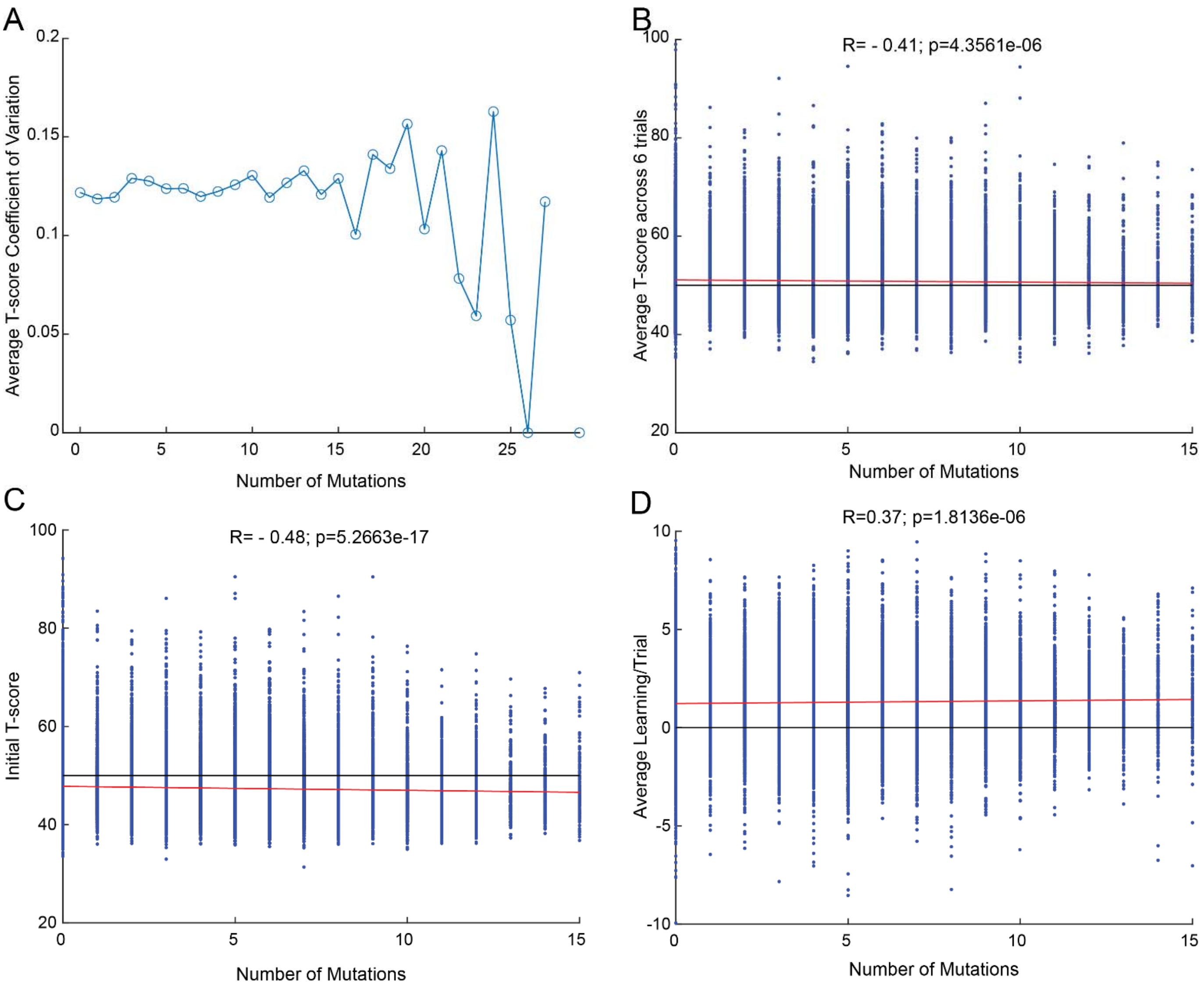

Impact of mutation rate on rotarod performance. A. Coefficient of variation of T-scores for a given number of homozygous mutations in a mouse. Note a relatively large negative change in variation after 15 mutations in a single mouse. B. Average T-score for each mouse represented against the number of homozygous mutations in that mouse. C. Initial T-score for each mouse plotted against the number of homozygous mutations in that mouse. D. Average learning per trial for each mouse plotted against the number of homozygous mutations in that mouse. Black horizontal lines are shown for comparison with the red best fit line.

bioRxiv preprint doi: https://doi.org/10.1101/2023.06.28.546756; this version posted June 30, 2023. The copyright holder for this preprint (which was not certified by peer review) is the author/funder, who has granted bioRxiv a license to display the preprint in perpetuity. It is made available under aCC-BY 4.0 International license.

Supplementary figure 4

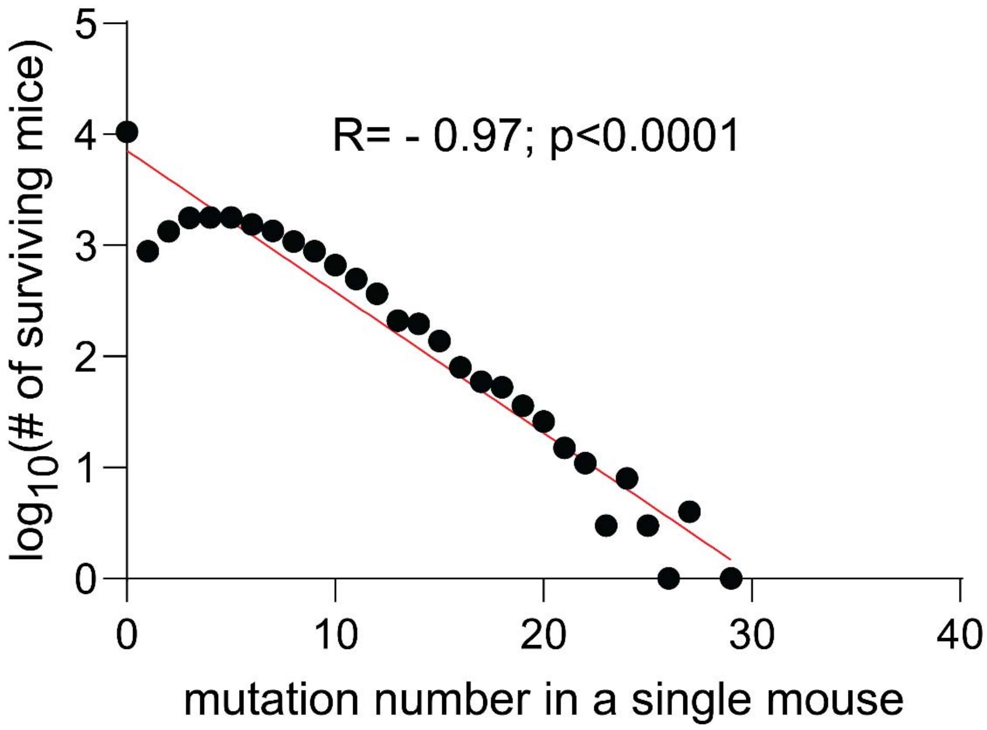

Mutation number in any single mouse in relation to the number of mice surviving with that mutation. R indicates the Pearson's correlation coefficient.
